# Forecast of the Future Trend of Accidents in an Electricity Distribution Company of Iran: A Time Series Analysis

**Published:** 2019-12

**Authors:** Alireza KHAMMAR, Seyednouredin HOSSEINIGHOSHEH, Anna ABDOLSHAHI, Mirmohammad HOSSEINI AHAGH, Mohsen POURSADEQIYAN

**Affiliations:** 1. Department of Occupational Health Engineering, Zabol University of Medical Sciences, Zabol, Iran; 2. Behbahan Faculty of Medical Sciences, Behbahan, Iran; 3. Food Safety Research Center (Salt), Semnan University of Medical Sciences, Semnan, Iran; 4. Department of Public Health, Khalkhal University of Medical Sciences, Khalkhal, Iran; 5. Health Management and Economics Research Center, Iran University of Medical Sciences, Tehran, Iran

## Dear Editor-in-Chief

Accidents are main problem of industries such as construction, electrical and other industries, which are led to fatal on non-fatal injuries is cause of accident in (1). However, many workers are exposed to electrical energy during their daily tasks, and some of them are unaware of the potential electrical hazards in the work environment. It is necessary to recognize the potential risk factors for electrical injuries especially fatal ones and to provide useful recommendations for developing effective safety programs to reduce the risk of electrocution (2). Although lots of technical preventive aspects are well known, electric accidents continue to occur (3). Considering the nature of accident data, the time-series model is a useful model in accident investigations. Time-series analyses are utilized in order to forecast future accident data based on past trends (4).

This study was conducted to model the accidents using the time series analysis in an electricity distribution company in Tehran, Iran. In phase one, data related to 2005 to 2012 were collected from the database of the safety department (5). Time Series analysis and Trend analysis were used for analyzing the data and forecasting the accidents up to 2017. The statistical calculations and analysis were performed using Minitab version 14 for time series model to forecast accidents among the workers of this company up to 2017.

**Fig. 1:**
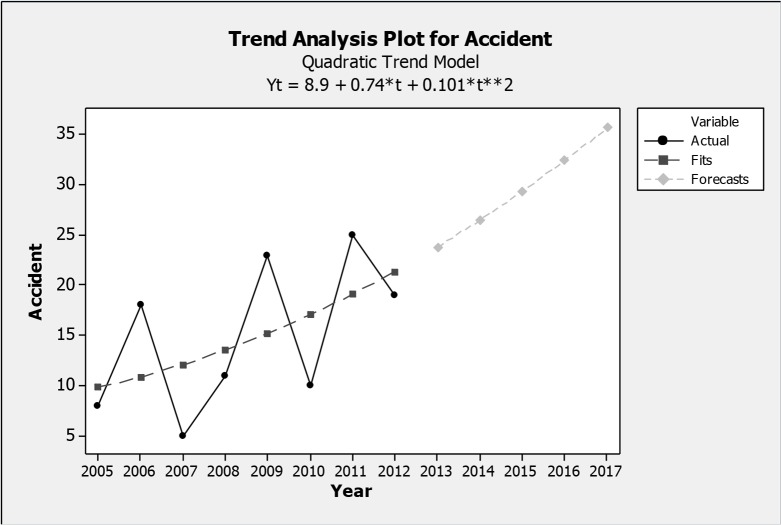
Trend analysis for accident forecasting during 2013–2017 based on accident trend during 2005–2012

**Fig. 2:**
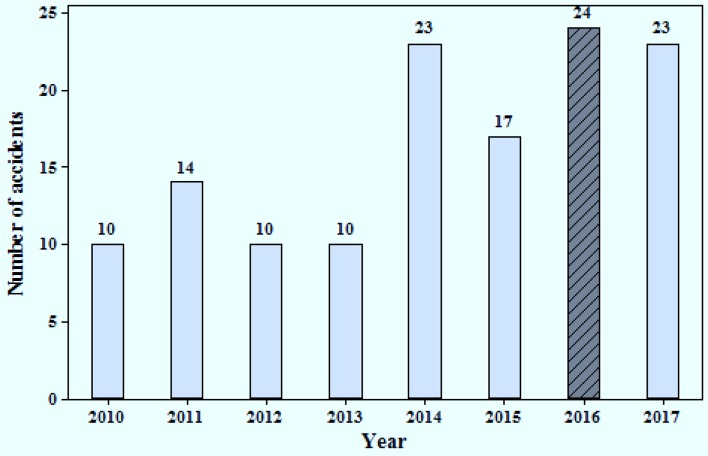
Frequency distribution of injured workers (from 2010 to 2017) (n=131)

Based on the analysis of the accidents in the first 8 years (2005 to 2012) reported in pervious study, 119 accidents were observed and an increasing trend was predicted in the model in Fig. 1. The gray dotted line indicates the fitted model and continuous line is related to existing data. Indeed, this graph shows a forecasting model for accidents in future based on past trend used to predict the rate of accidents during 2013–2017 (dotted line related to these years), and, by assuming the continuation of this trend, there will be more than 35 accidents per year up to 2017. In the second step, we tried to revise and categorize the registered data in order to get more precise information. Therefore, the number of accidents was reduced compared with the previous data (such as the years of 2011 and 2012). Therefore, regarding the accuracy of available data, we surveyed 131 occurred accident from 2010 to 2017 (there was no data for updating before 2010). The final results are drawn in accordance with the incremental trend in Fig. 2.

The results obtained from this study showed an increasing trend of accidents which is consistent with the study conducted in Yazd on construction accidents (6). Another study which applied the time-series analyses to predict the future accident data based on the resisted trends in past showed an increasing trend of injuries and accidents especially in June (52 accident) which even was 40% higher than the number of occurred accidents in June 1987 (4).

However, the mean of the frequency distribution of injured workers during 2010 to 2017 was approximately 20 and model predicted 35 cases of accidents which this difference can be due to taking some engineering and administrative control such as training course for improving safety behavior to reduce the incidence of accidents (7, 8). This aim is ascertainable whit risk assessment and rise level of safety climate (9, 10).
